# Polycomb Group Protein Ezh2 Regulates Hepatic Progenitor Cell Proliferation and Differentiation in Murine Embryonic Liver

**DOI:** 10.1371/journal.pone.0104776

**Published:** 2014-08-25

**Authors:** Hiroyuki Koike, Rie Ouchi, Yasuharu Ueno, Susumu Nakata, Yuta Obana, Keisuke Sekine, Yun-Wen Zheng, Takanori Takebe, Kyoichi Isono, Haruhiko Koseki, Hideki Taniguchi

**Affiliations:** 1 Department of Regenerative Medicine, Yokohama City University Graduate School of Medicine, Kanazawa-ku, Yokohama, Kanagawa, Japan; 2 Division of Oncological Pathology, Aichi Cancer Center Research Institute, Chikusa-ku, Nagoya, Japan; 3 Project Leader of Advanced Medical Research Center, Yokohama City University, Kanazawa-ku, Yokohama, Kanagawa, Japan; 4 PRESTO, Japan Science and Technology Agency, Saitama, Japan; 5 Laboratory for Developmental Genetics, RIKEN Research Center for Allergy and Immunology, Tsurumi-ku, Yokohama, Kanagawa, Japan; Institute of Hepatology, Foundation for Liver Research, United Kingdom

## Abstract

In embryonic liver, hepatic progenitor cells are actively proliferating and generate a fundamental cellular pool for establishing parenchymal components. However, the molecular basis for the expansion of the progenitors maintaining their immature state remains elusive. Polycomb group proteins regulate gene expression throughout the genome by modulating of chromatin structure and play crucial roles in development. *Enhancer of zeste homolog 2* (*Ezh2*), a key component of polycomb group proteins, catalyzes tri-methylation of lysine 27 of histone H3 (H3K27me3), which trigger the gene suppression. In the present study, we investigated a role of *Ezh2* in the regulation of the expanding hepatic progenitor population *in vivo*. We found that Ezh2 is highly expressed in the actively proliferating cells at the early developmental stage. Using a conditional knockout mouse model, we show that the deletion of the SET domain of *Ezh2*, which is responsible for catalytic induction of H3K27me3, results in significant reduction of the total liver size, absolute number of liver parenchymal cells, and hepatic progenitor cell population in size. A clonal colony assay in the hepatic progenitor cells directly isolated from *in vivo* fetal livers revealed that the bi-potent clonogenicity was significantly attenuated by the Ezh2 loss of function. Moreover, a marker expression based analysis and a global gene expression analysis showed that the knockout of Ezh2 inhibited differentiation to hepatocyte with reduced expression of a number of liver-function related genes. Taken together, our results indicate that Ezh2 is required for the hepatic progenitor expansion *in vivo*, which is essential for the functional maturation of embryonic liver, through its activity for catalyzing H3K27me3.

## Introduction

Stem cells are defined as populations that generate and maintain whole cell populations in a tissue and organ, which have capacity of self-renewal and differentiation to multi-lineage progenitor cells. Stem cells in the hematopoietic system, for instance, remain basically in quiescent state [1], in contrast to progenitor cells that actively proliferate and subsequently differentiate to mature cells [2]. In fetal mouse liver, we previously reported the hepatic stem/progenitor cells can be isolated in the fraction of c-kit^−^ CD49f^+/low^ CD29^+^ CD45^−^ TER119^−^ cells, which have marked proliferative capacity and bi-potency for differentiation into both hepatocyte and cholangiocyte [3]. However, molecular mechanisms underlying the active proliferation of the hepatic progenitor cells maintaining their immature state are yet to be elucidated.

Polycomb group (PcG) proteins constitute transcriptional repressor machineries via changing chromatin structures [4], [5]. PcG form two major distinct protein complexes: polycomb repressive complex (PRC) 1 and PRC2. Ezh2, a PcG protein homologous to *Drosophila enhancer of zeste*, is a component of PRC2 and has a SET domain that catalyzes tri-methylation of lysine 27 on histone H3 (H3K27me3) [5]. Ezh2 first marks H3K27me3 on target genes, which lead to the recruitment of PRC1, and the two PRCs cooperate in the repression of the genes. In *Drosophila*, PcG is established as the repressor of hox genes, a set of transcription factors that specify cell identities [6]. In mammals, an increasing body of evidence suggests that PcG plays crucial roles in regulation of the embryonic stem (ES) cells and somatic stem cells [7]–[10].

Ezh2 regulates expression of target genes in the genome, which differ in different tissues and organs [11]. It is reported that Ezh2 is essential for regulation of ES cells [12]. H3K27me3, a repressive histone modification induced by Ezh2, marks promoters of the developmental regulator genes overlapping with an active histone modification of H3K4me3, so-called bivalent domains, to keep the developmental regulators poised [13]. In a study of induced pluripotent stem cells (iPSCs), it was shown that Ezh2 is not only able to increase efficiency of iPSC generation, but can also be used as part of a new reprogramming cocktail with other transcription factors [14]. It has been shown that Ezh2 is tightly involved also in the regulation of somatic stem/progenitor cells [15], [16]. Interestingly Ezh2 is involved in fetal hematopoietic [16] and epidermal [17] stem cells, rather than in the adult stem/progenitor counterparts. Besides Ezh2, Ezh1, which also induces tri-methylation at H3K27, has been reported as a critical regulator for adult hematopoietic stem/progenitor cells [18]. These findings suggest that the process of gene expression pattern by PcG may change dynamically from fetal to adult individuals. However, little is known about the function of Ezh2 in the regulation of the hepatic stem/progenitor cells in embryonic liver.

A previous study showed that a shRNA-based knockdown of *Ezh2* inhibited proliferation of cultured hepatic progenitor cells *in vitro* and induced expression of hepatic differentiation marker genes, suggesting a blocking effect of Ezh2 for differentiation [19]. In liver development *in vivo*, however, it has not been tested if *Ezh2* actually regulates proliferation of hepatic progenitor population and differentiation. As early embryonic lethality of *Ezh2* knockout mice by ED 7.5 has impeded elucidation of Ezh2 function in liver development, we employed a conditional knockout mouse model, inducing deletion of a SET domain in *Ezh2* that catalyzes tri-methylation of H3K27, upon tamoxifen (TAM) administration. In the present study, we show that the conditional knockout of *Ezh2* functional domain causes significant blockade of liver growth. Ezh2 function is essential for expansion of hepatic progenitor population, and its loss of function results in decreased expression of hepatic differentiation marker genes and also functional genes for liver.

## Materials and Methods

### Mice

Pregnant C57BL/6 mice were purchased from Japan SLC (Japan). Ezh2^F/F^ mice were crossed with Rosa26::CreER(T2)^+/−^ mice [16]. For conditional deletion of Ezh2, Ezh2^F/F^ mouse had alleles in which exons 18 and 19 encoding the SET catalytic domain were flanked with loxP sequences. To induce CreER(T2) activity, mice were injected with 4-hydroxy tamoxifen (TAM; Sigma-Aldrich, Switzerland) at a dose of 1 mg/body intraperitoneally for 3 consecutive days. Mice were bred and maintained in the Animal Research Facility of the Graduate School of Yokohama City University in accordance with institutional guidelines. All animal experiments in this study were performed under approval from the institutional animal care and use committee of Yokohama City University (Permit Number: 11-64).

### Genomic PCR

Genotype of Rosa26::CreER(T2)^+/−^ Ezh2^F/F^ fetal mice was confirmed with extracted genomic DNA from their limbs. PCR reaction was performed by Fast Cycling PCR kit (Qiagen, Germany). Primer sequences for CreER(T2)^+/−^ were listed in Table S1.

### Preparation of fetal liver cells

Livers were acquired from fetal mice at embryonic day (ED) 11.5, 13.5, 15.5, and 17.5 of timed pregnant mice, and CreER(T2)^+/−^ Ezh2^F/F^ (*Ezh2 SET domain* depleted) and CreER(T2)^−/−^ Ezh2^F/F^ (the control) fetal mice at ED 13.5 (TAM; ED 8.5–10.5) and 18.5 (TAM; ED 10.5–12.5). The livers were dissociated by incubating with 0.2% trypsin–washing medium (DMEM/F12 containing 5% fetal bovine serum) on ice for 30 minutes and shaking at 37°C for 15 minutes. After pipetting and wash, cells were triturated and passed through 40 µm nylon meshes to obtain a single-cell suspension.

### Isolation of non-hematopoietic liver parenchymal cells

Fetal liver cells were incubated with biotin-conjugated anti-TER119 (BD Biosciences) and biotin-conjugated anti-CD45 (BD Biosciences) antibodies on ice for 30 min. After wash, cells were reacted with Streptavidin Particles Plus (BD Biosciences) on ice for 30 min. The reacted sample was added into the 2 mL IMag buffer (PBS containing 0.5% BSA and 2 mM EDTA), and TER119^+^/CD45^+^ hematopoietic cells were removed by a Cell Separation Magnet (BD Biosciences).

### Western blot analysis

Fetal TER119^−^ CD45^−^ liver cells were washed and lysed in 10 mM Tris (pH 7.5), 150 mM NaCl, 1% NP40, and protease inhibitor cocktail (Roche Applied Science) on ice for 30 min. Ten µg of protein were separated by SDS-PAGE, transferred to poly-vinylidene fluoride (PVDF) membrane. After blocking with 5% skim milk in PBST for an hour at room temperature, the membrane was incubated with antibodies to Ezh2 (1∶200), Bmi1 (1∶500), Ring1B (1∶200), albumin (1∶200), β-actin (1∶1000) over night at 4°C, which are listed in Table S2. Molecular weight of Ezh2, Bmi1, Ring1B, albumin, β-actin are 98, 44, 40, 70, and 42 kDa, respectively. After wash, the membrane was incubated for 1 hour at room temperature with HRP-conjugated anti mouse IgG (GE healthcare). Further washing, the membrane was incubated with ECL Western Blot Detection regents, and chemiluminescent images were collected on a LAS3000 (Japan).

### Immunofluorescence staining

Liver tissues of fetal mice were embedded in Tissue-Tek OCT compound 4583 (Sakura Finetechnical, Japan) in liquid nitrogen and sectioned at 5 µm. Cultured cells were washed with 1× PBS and performed as followings. Each sample of tissues and cultured cells was fixed by aceton/methanol at −30°C for 10 minutes. Nonspecific binding was blocked with 10% goat serum for an hour at room temperature. The samples were incubated with primary antibodies at 4°C overnight, which are listed in Table S2. After wash, cells were incubated with secondary antibodies (Molecular Probes, OR, USA) matched with each primary antibody for 1.5 hours at room temperature. After final wash, the samples were mounted with medium containing DAPI (Vector Laboratories, CA, USA), and viewed using an Axio Imager scanning microscope (Carl Zeiss, Germany), and the Leica TCS SP5 confocal microscope.

### BrdU Staining

To detect rapidly dividing cells in liver, bromodeoxyuridine (BrdU; Sigma-Aldrich) at a dose of 0.5 mg/body was administered maternal intraperitoneally. Fetal mice were subsequently sacrificed 1 hour later. For detection of BrdU incorporated into DNA, sections of liver tissues were incubated in 2 N HCl at 37°C for 30 min and 0.1 M borate buffer (pH 8.5) for 15 minutes at room temperature. The staining was performed as described in the above *immunofluorescence staining* section with anti-BrdU antibody (BD Biosciences, CA, USA).

### Quantitative real-time polymerase chain reaction (qRT-PCR)

Total RNA of fetal mouse TER119^−^ CD45^−^ liver cells and cultured hepatic progenitor cells were extracted using Isogen reagent (Nippon Gene, Japan) according to the manufacturer's protocol. cDNA was synthesized from 1 µg of total RNA using the high capacity cDNA reverse Transcription Kit (Applied Biosystems, CA, USA). Quantitative RT-PCR was performed with the Roche Universal Probe system and the Eagle Taq Master Mix (Roche Applied Science, Germany). Information of all primers is shown in Table S3.

### Hematoxylin/eosin and Cdkn1a-immunohistochemical staining

Liver tissues of fetal mice were fixed with 4% PFA at 4°C for overnight, processed, and embedded in paraffin. Sections (5 µm) were placed on MAS-coated slides for standard histological staining with hematoxylin/eosin (HE). For Cdkn1a staining, antigen retrieval was performed in heated Tris-EDTA buffer (pH 9) with microwave for 15 min. The blocked samples with 10% normal goat serum were incubated with anti-Cdkn1a antibody (1∶25) for overnight at 4°C. As secondary antibody, EnVision^+^ kit (DAKO) was used, and signals were visualized with AEC^+^ substrate (DAKO), according to the manufacturers instruction.

### Microarray and data analysis

Total RNA of fetal liver excluded hematopoietic cells was isolated using TRIzol Reagent (Life Technologies, CA, USA) and. For quality control of total RNA samples, Agilent 2100 Bioanalyzer was used. Expression profiling was obtained with Whole Mouse Genome 4x44K v2 OligoDNA Microarray Kit (Agilent Technologies) according to manufacturer's instruction. Hybridization signals were scanned and processed by 75% percentile shift normalization using GeneSpring GX11.5.1 software. A hierarchical clustering method with Euclidean distance complete linkage was used for creation of the heat map to compare expression of the liver development signature gene set, which was previously shown to be expressed in parallel with progression of liver development [20]. For the corrected p-values in the Gene Ontology Term Enrichment analysis, the Benjamini and Yekutieli correction method was applied. The pathway analysis on up-regulated genes with 2- and 8-fold changes by the Ezh2 KO was performed with the WikiPathways database (http://www.wikipathways.org) using the GeneSpring software. All raw data of microarray analyses in this study have been deposited in the Gene Expression Omnibus database (Accession Number: GSE46631, GSE54029)

### Fluorescence activated cell sorting

Single cells suspension of fetal liver was incubated in a 96 well plate (Nunc) on ice for 30 minutes with monoclonal antibodies, which are listed in Table S2. After washing, cells were incubated with streptavidin-conjugated APC-Cy7 florescent probe on ice for 20 min. The fluorescence-labeled cells were analyzed, and c-kit^−^ CD49f^+/low^ CD29^+^ TER119^−^ CD45^−^ cells were separated with Flowcytometer MoFlo (Dako Cytomation, Denmark; Summit version 4.0) after excluding PI^+^ dead cells.

### Clonogenicity assay

The freshly sorted cells were plated and cultured on type IV collagen-coated 6-well plates (BD Biosciences) at a density of 2.5×10^3^ cells/cm^2^. Our standard culture medium for the primary hepatic progenitor cells was described previously [3]. During the culture period, cells were incubated at 37°C in a humidified atmosphere of 5% CO_2_. Images of immunostained colonies were obtained and analyzed by Axio scope fluorescent microscope (Carl Zeiss) and IN Cell Analyzer 2000 (GE Healthcare, UK)

### Chromatin immunoprecipitation assay

CD45^−^ TER119^−^ non-hematopoietic liver cells at ED 13.5 were cross-linked using formaldehyde (final concentration of 1%) at room temperature for 20 minutes. The fixed cells were washed with ice-cold PBS and then harvested in lysis buffer and left on ice for 10 minutes. Nuclear pellets were disrupted using a sonicator (BioRuptor), yielding genomic DNA fragments with a bulk size of 100–300 bp. After centrifugation, the supernatant was transferred to a new tube. The cell lysate was diluted before antibody addition to reduce the amount of complexes brought down nonspecifically by the 50% protein Sepharose beads in a subsequent step. A primary antibody, which are listed in Table S2, was added to the lysate, and the mixture was incubated overnight at 4°C. Immune complexes were recovered by adding blocked-protein A beads and incubating for 2 h at 4°C. The beads were washed 2 times with 1 mL of wash buffer, LiCl buffer, and two times with tris–EDTA (TE) buffer (pH 7.5) and eluted with elution buffer at 65°C overnight. The eluted material was incubated with proteinase K at 37°C for 2 hours, phenol/chloroform-extracted and finally ethanol-precipitated. The resulting DNA was dissolved in 30 µL of TE. The primers were used to amplify genomic sequences including a start point of transcription of CDKIs, which are indicated in Table S4. Each PCR reaction generated only the expected specific amplicon as verified by gel electrophoresis of the PCR products.

## Results

### Ezh2 is highly expressed in parenchymal cells of embryonic liver in earlier stages

**Figure 1:**
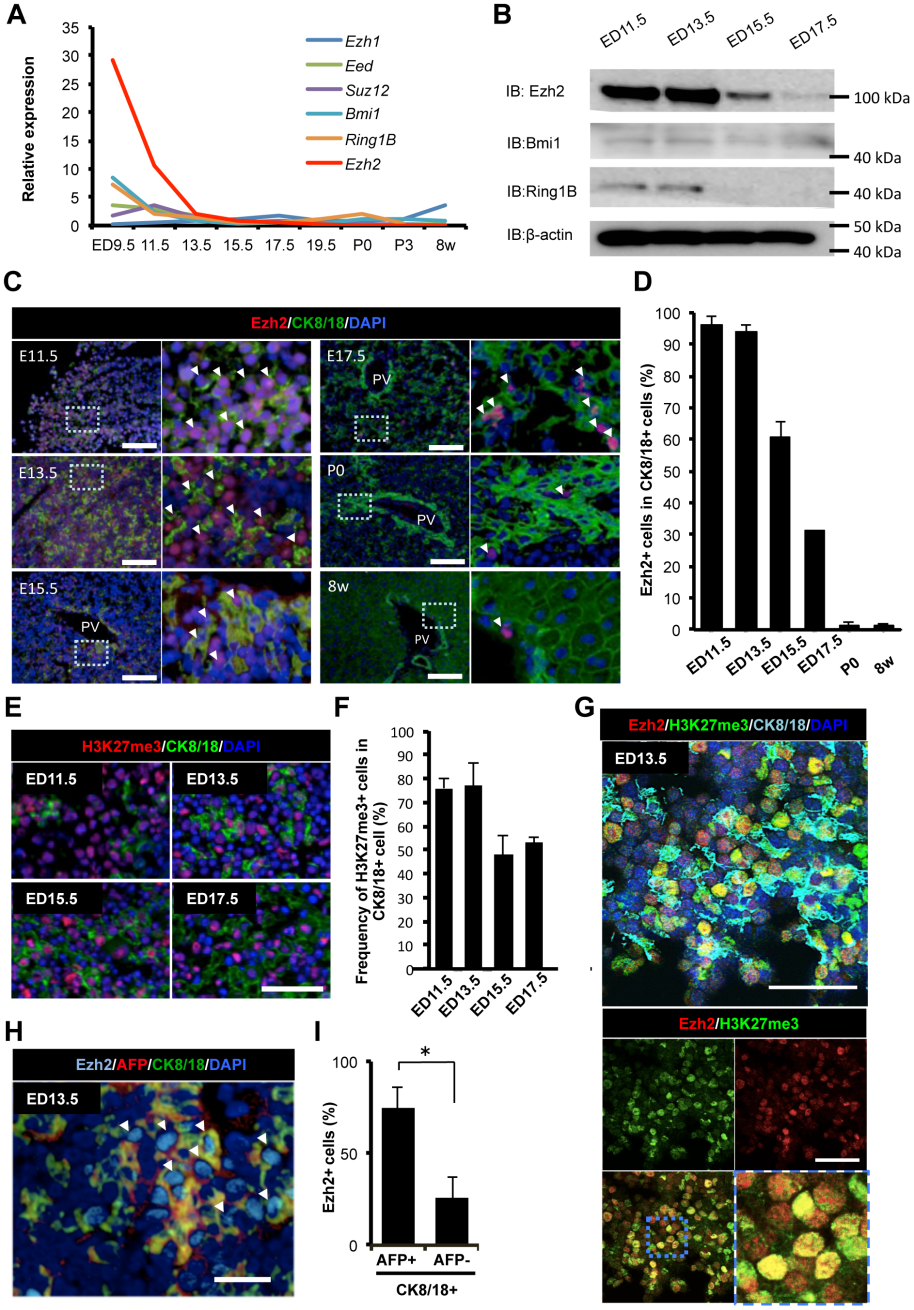
Ezh2 is highly expressed in parenchymal epithelial cells in early developing liver. A: Time course analysis of expression levels of the polycomb group (PcG) members (Ezh1, Eed, Suz12, Bmi1, Ring1B, and Ezh2) in CD45^−^ TER119^−^ non-hematopoietic liver cells of mice at nine time points between ED 9.5 and 8 weeks. Relative expression values from microarray analyses are shown. B: Western blot analysis of Ezh2 in CD45^−^ TER119^−^ non-hematopoietic liver cells of fetal mice at ED 11.5, 13.5, 15.5, and 17.5. β-actin is indicated as control. C: Immunofluorescence staining for Ezh2, CK8/18 (epithelial marker), and DAPI in liver of fetuses at ED 11.5, 13.5, 15.5, 17.5, P 0 (neonatal), and 8 weeks. Arrows indicate Ezh2 positive cells in CK8/18 positive cells. (Scale bar: 100 µm) D: Frequency of Ezh2 positive cells in CK8/18 positive cells at indicated time points. (n = 3). E: Immunofluorescence staining for histone H3K27me3, CK8/18 (epithelial marker), DAPI in the liver tissues at ED 11.5, 13.5, 15.5, and 17.5. Scale bar = 100 µm. F: Frequency of H3K27me3 expressing cells in CK8/18 positive cells at indicated points. Data are mean ± SD (n = 3). G Immunofluorescence staining for Ezh2, H3K27me3, Ck8/18 and DAPI in the liver of fetuses at ED 13.5. Upper is merge image of all colors. Bottom column shows image of Ezh2 and H3K27me3, lower right is magnified image of left. Red: Ezh2, Green: H3K27me3, Light blue: CK8/18, Blue: DAPI. Scale bar = 100 µm. H Immunofluorescence staining for AFP, Ezh2, CK8/18, and DAPI in the liver of fetuses at ED 13.5. Arrows indicate AFP and Ezh2 double positive cells in CK8/18 positive cells. (Scale bar: 50 µm) I: Frequency of Ezh2 positive cells in AFP and CK8/18 double positive cells and AFP negative and CK8/18 positive cells at indicated time points. (n = 3). *P* values (asterisks) are from the Mann–Whitney *U*-test. *P<0.05.

We first examined the expression of polycomb group (PcG) genes; *Ezh1*, *Eed*, *Suz12*, *Bmi1*, *Ring1B* and *Ezh2* in the liver development of mice. Most of the PcG genes tested were expressed at the early liver development at ED 9.5 and 11.5 and decreased after ED 13.5 (Figure 1A). Among the PcG genes, the expression level of *Ezh2* was significantly high at ED 9.5 and 11.5 (Figure 1A). To focus on the expression of the Ezh2 protein in the liver development, western blot and immunofluorescence analyses were performed. A western blot analysis revealed that the Ezh2 protein is highly expressed in the liver cells at ED 11.5 and 13.5 and gradually decreased along with the progression of the development (Figure 1B). Ring1B protein levels exhibit higher expression in ED 11.5–ED 13.5, similar to Ezh2, but signals of Ring1B were relatively weak. In contrast, we did not observe clear change in protein levels of Bmi1, and signals were faint at every time point, in line with the results at the mRNA levels (Figure 1B). Furthermore, an immunofluorescence analysis showed that Ezh2 positive cells were enriched in 93% and 95% of cytokeratin (CK) 8/18 positive epithelial cells at the early stage but decreased along with the progression of the development (Figures 1C and 1D). Significantly high levels of the tri-methylation of H3K27 were also detected in the early stage compared with in the late stage (Figures 1E and 1F). Co-immunofluorescence staining of Ezh2 and H3K27me3 in liver tissues at ED13.5 revealed that majority of CK8/18-positive epithelial cells express both Ezh2 and H3K27me3 (Figure 1G). These results suggest that Ezh2 would play a pivotal role particularly in the embryonic liver.

To verify the frequency of Ezh2 positive cells in hepatic lineage-committed immature cells, we next performed double-immunofluorescence staining of alpha fetoprotein (AFP), a marker for undifferentiated hepatic cells, together with Ezh2. Ezh2 positive cells were observed in 74.5% of AFP positive cells in the livers at ED 13.5 while Ezh2 positive cells were detected only in 25.5% of AFP negative cells. (Figures 1H and 1I). These results demonstrated that Ezh2 is highly expressed in AFP-positive epithelial cells in early steps of fetal liver development in mice.

### Ezh2 SET domain depletion impaired growth of embryonic livers

**Figure 2:**
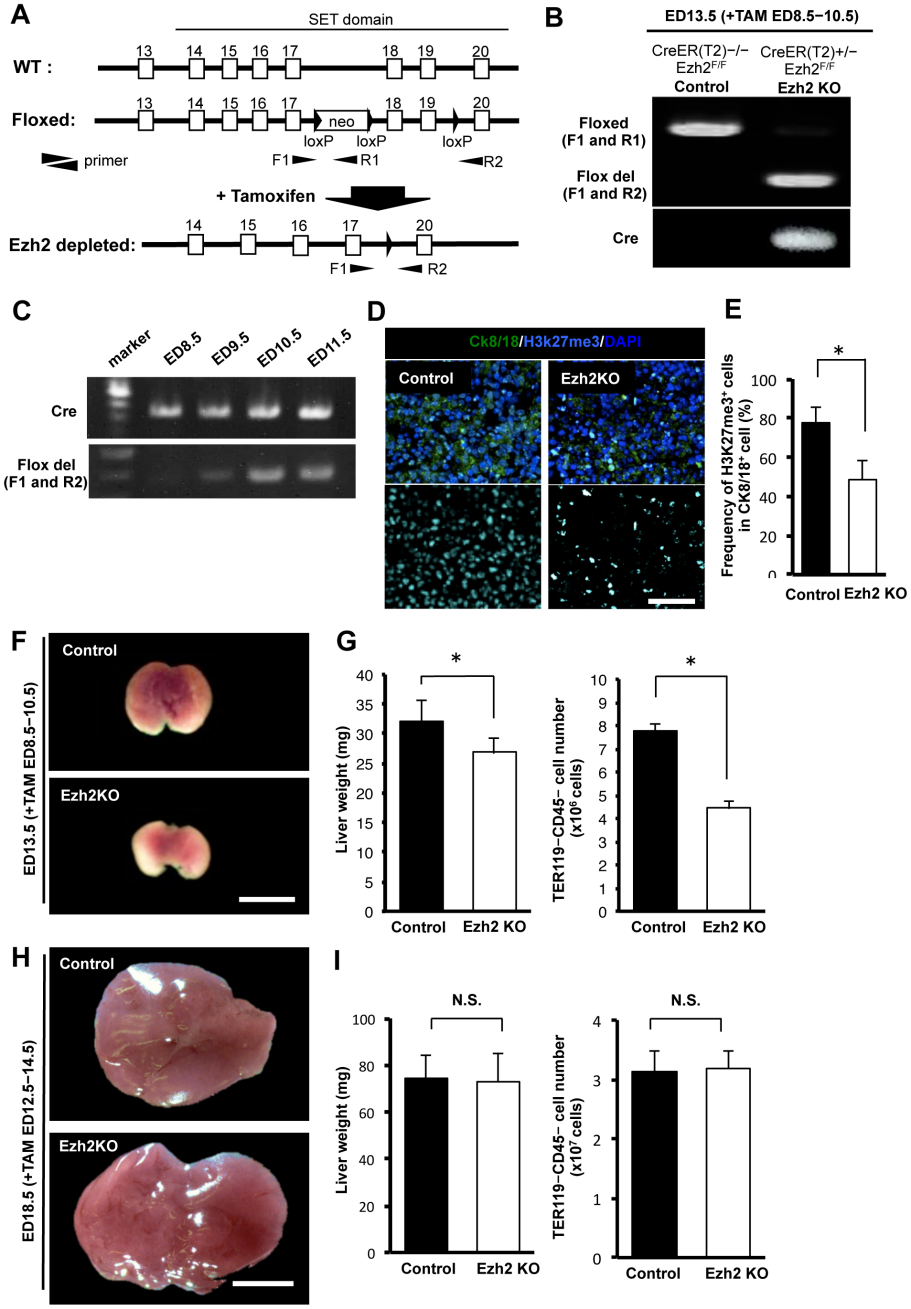
Ezh2 SET domain depletion impaired liver growth. A: Schematic diagram of *Ezh2* SET domain KO system. Black triangle, square, and black narrow triangle are indicted as loxP sequence, exon, and primers respectively. F1, R1, and R2 mean forward primer 1, reverse primer 1 and 2. B: Deletion of Ezh2 in liver cells of fetal mouse ED 13.5 obtained from pregnant mouse injected with tamoxifen (TAM) at ED 8.5–10.5 detected by genomic PCR. Cre: Cre allele; Floxed: Floxed *Ezh2* allele; and Flox del: floxed *Ezh2* allele after removal of exons 18 and 19 by Cre recombinase, respectively. C: Efficiency of deletion of Ezh2 SET domain in fetal mouse at ED 8.5, 9.5, 10.5, and 11.5 (TAM administrated from ED 8.5) by genomic PCR. The bands for Ezh2 SET domain depletion (Flox del) appeared completely clear at ED 10.5. D: Immunofluorescence staining for H3K27me3, CK8/18 (epithelial marker) and DAPI in the liver tissues of control and Ezh2 KO at ED 13.5 after 3 days TAM injection (ED 8.5–10.5). E: frequency of H3K27me3 expressing cells in CK8/18 positive cells at indicated points. Data are mean ± SD (n = 3). *P* values (asterisks) are from the Mann–Whitney *U*-test. *P<0.05. F: Macroscopic images of livers in the control (CreER(T2)^−/−^;Ezh2^F/F^ mouse) and Ezh2 KO (Ezh2 SET domain depleted mouse; Rosa26::CreER(T2)^+/−^;Ezh2^F/F^ mouse) at ED 13.5 after 3 days TAM injection (ED 8.5–10.5). Scale bar = 100 µm. G: Left panel: liver weight of the control and Ezh2 KO at ED 13.5 (TAM; ED 8.5–10.5). Right panel: Absolute number of TER119^−^ CD45^−^ liver cells per a liver of the control and Ezh2 KO at ED 13.5 (TAM; ED 8.5–10.5). Data are mean ± SD (n = 3). *P* values (asterisks) are from the Mann–Whitney *U*-test. *P<0.05. H: Appearance of livers in the control and Ezh2 KO at ED 18.5 after 3 days TAM injection (ED 12.5–14.5). Scale bar = 100 µm. I: Left panel: liver weight of the control and Ezh2 KO at ED 18.5 (TAM; ED 12.5–14.5). Right panel: Absolute number of TER119^−^ CD45^−^ cells per a liver of the control and Ezh2 KO at ED 18.5 (TAM; ED 12.5–14.5). Data are mean ± SD (n = 3). *P<0.05. N.S.; not significant (*P*>0.05).

To investigate the regulatory role of Ezh2 in embryonic liver *in vivo*, we used systemic *Ezh2* conditional knockout (CreER(T2)^+/−^ Ezh2^F/F^) mice, in which depletion of Ezh2 SET domain, the catalytic domain for tri-methylation of H3K27, can be induced by tamoxifen administration (Figures 2A and 2B). To deplete the Ezh2 SET domain at early stage when hepatic progenitor cells are actively proliferating, we administrated tamoxifen from ED 8.5 and examined the liver at ED 13.5. A genomic PCR confirmed that Ezh2 SET domain (exon 18 to 19) was completely depleted at ED 10.5 in the CreER(T2)^+/−^ Ezh2^F/F^ mice (Figure 2C). As expected, an immunofluorescence analysis showed that frequency of H3K27me3 positive cells were significantly decreased in Ezh2 SET domain depleted CreER(T2)^+/−^ Ezh2^F/F^ liver at ED 13.5 (Figure 2D and 2E).

Macroscopically, the depletion of Ezh2 SET domain at early stage resulted in apparent reduction in liver size at ED 13.5 in CreER(T2)^+/−^ Ezh2^F/F^ fetal mice compared to those in CreER(T2)^−/−^ Ezh2^F/F^ control mice (Figure 2F and Figure S1). The weight of CreER(T2)^+/−^ Ezh2^F/F^ fetal livers was remarkably decreased than that of the control (Figure 2G). Moreover, number of TER119^−^ CD45^−^ non-hematopoietic cells obtained from CreER(T2)^+/−^ Ezh2^F/F^ fetal liver was decreased by 57.4% compared to the control (Figure 2G). However, when we induced Ezh2 SET domain depletion at the late stages (TAM; ED 12.5–14.5) when the hepatic progenitor cells rarely existed, no significant difference was observed in liver size, weight, and frequency of TER119^−^ CD45^−^ non-hematopoietic cells, between CreER(T2)^+/−^ Ezh2^F/F^ and CreER(T2)^−/−^ Ezh2^F/F^ livers at ED 18.5 (Figures 2H and 2I).

### Negative regulators of cell cycle were significantly up-regulated in the Ezh2 SET domain depleted fetal livers

**Figure 3:**
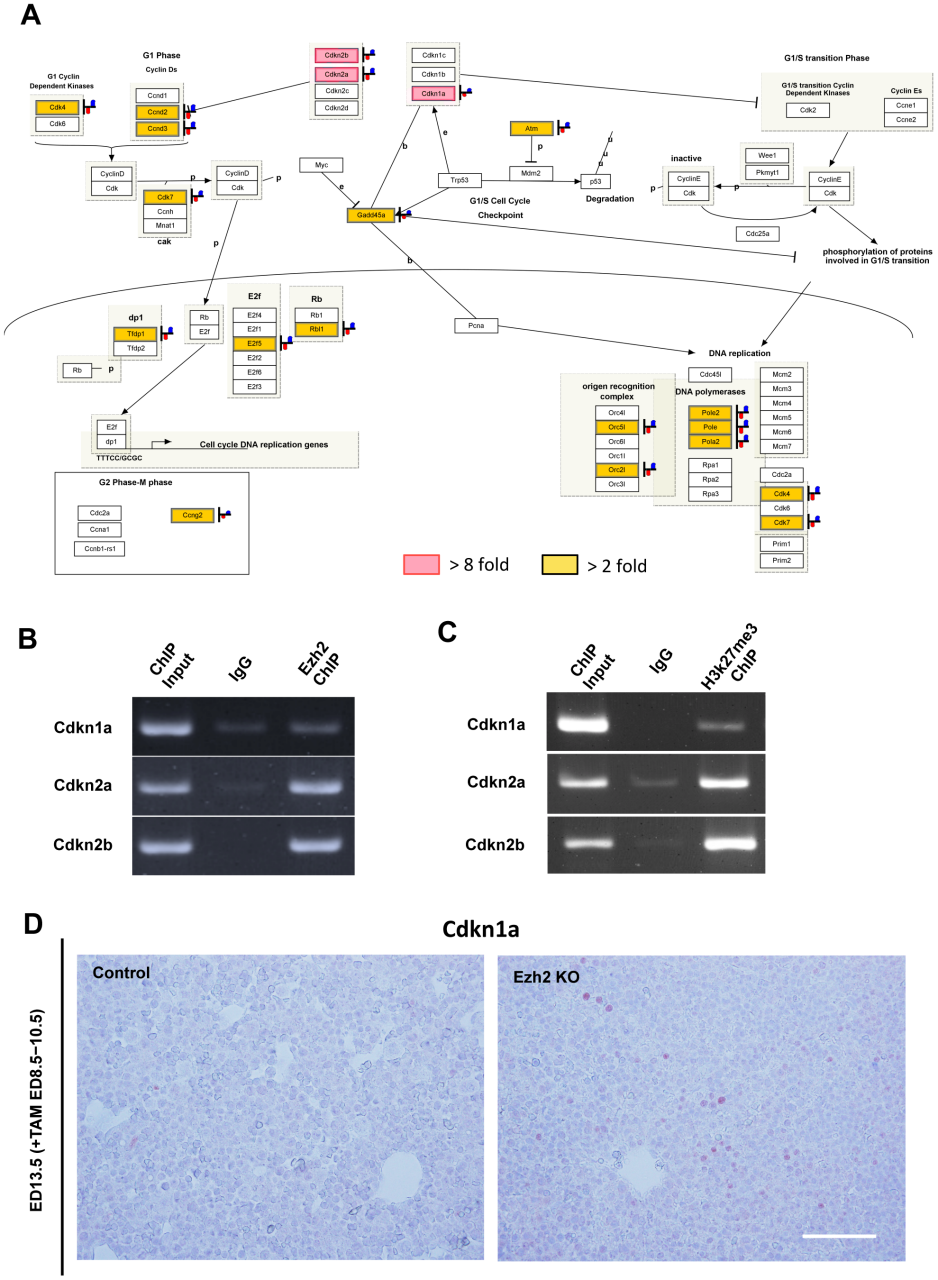
Negative regulators of cell cycle were significantly up-regulated in the Ezh2 SET domain depleted fetal livers. A: The G1_to_S_Cell_Cycle_Control pathway (WP413_41269) from analysis using the WikiPathways platform is shown, in which gene hits with more than 2-fold change up to 8-fold change (*P* = 0.0018) are highlighted in yellow and hits with more than 8-fold change (*P* = 0.0046) in red in the Ezh2-KO CD45^−^ TER119^−^ liver cells at ED 13.5 compared with WT control. B: ChIP-PCR analysis with the anti-Ezh2 antibody and the primer sets detecting promoter regions of Cdkn1a, Cdkn2a, and Cdkn2b in the CD45^−^ TER119^−^ liver cells from WT livers at ED 13.5 are shown. C: ChIP-PCR analysis with the anti-H3K27me3 antibody and the primer sets detecting promoter regions of Cdkn1a, Cdkn2a, and Cdkn2b in the CD45^−^ TER119^−^ liver cells from the WT control livers at ED 13.5 are shown. D: Immunohistochemical analysis of Cdkn1a protein in the WT control and Ezh2 KO liver tissues at ED 13.5. Scale bar = 50 µm.

To gain insights into mechanisms underlying the observed impairment of early liver development caused by Ezh2 SET domain depletion, we analyzed genes that were differentially expressed between control and KO liver cells at ED 13.5. The pathway analysis upon up-regulated genes by the Ezh2 SET domain depletion identified the cell cycle regulation- related pathway as a significantly altered category (Figure 3A and Table S5 and S6). Since Cdkn1a, Cdkn2a, and Cdkn2b expression were the most strongly induced among them, we studied whether promoter regions of the genes are accompanied with accumulation of Ezh2 and H3K27me3. ChIP assay with anti-Ezh2 antibody revealed direct interaction of the Ezh2 protein with the promoter regions of Cdkn1a, Cdkn2a, and Cdkn2b (Figure 3B). Indeed, accumulations of H3K27me3 repressive modification were confirmed with the promoter regions of Cdkn1a, Cdkn2a, and Cdkn2b (Figure 3C). Furthermore, protein expression of Cdkn1a was only detected within the Ezh2 KO livers whereas it was negative in the control liver at ED 13.5 (Figure 3D). These results suggest that the Ezh2KO-induced blockade of liver growth before ED 13.5 was accompanied with altered expression levels of the cell cycle regulating genes, and that de-repression of the genes such as Cdkn1a, Cdkn2a, and Cdkn2b would play important roles in the phenotype.

### Ezh2 SET domain depletion inhibited the proliferation of hepatic progenitor cells

**Figure 4:**
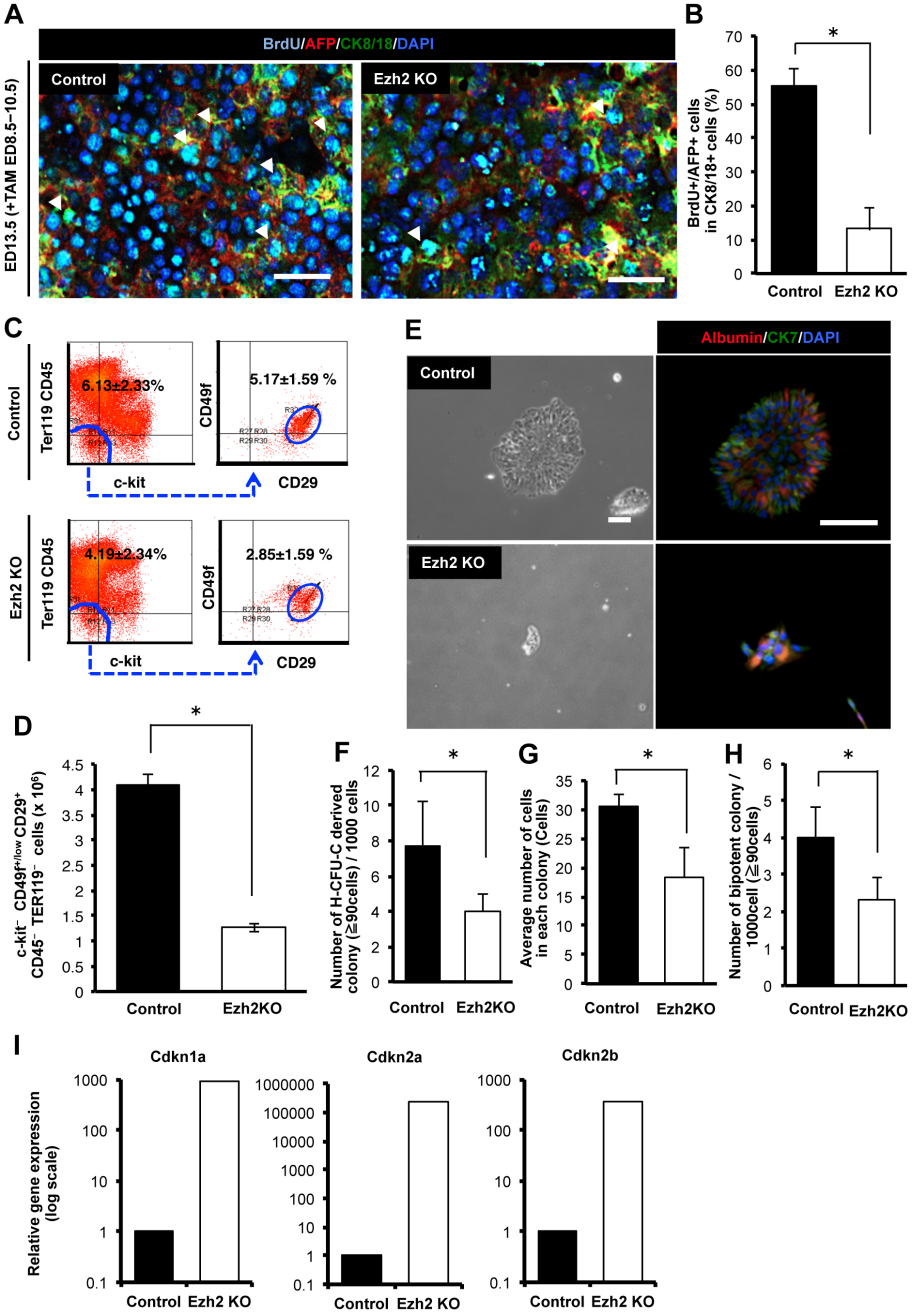
Ezh2 SET domain depletion inhibited the proliferation of hepatic progenitor cells. A: Immunofluorescence staining for AFP (hepatic undifferentiated marker), BrdU, CK8/18 (epithelial marker) and DAPI in the liver tissues of control (Rosa26::CreER(T2)^−/−^;Ezh2^F/F^ mouse) and Ezh2 KO (Ezh2 SET domain depleted mouse; Rosa26::CreER(T2)^+/−^;Ezh2^F/F^ mouse) at ED 13.5 after 3 days TAM injection (ED 8.5–10.5). Arrows indicates AFP and BrdU double positive cells in CK8/18 positive cells. Scale bar = 100 µm. B: Frequency of AFP and BrdU double positive cells in CK8/18 positive cells of the control and Ezh2 KO at ED 13.5 (TAM; ED 8.5–10.5). Data are mean ± SD (n = 3). *P* values (asterisks) are from the Mann–Whitney *U*-test. *P<0.05. C: Flowcytometric analysis of c-kit^−^ CD29^+^ CD49f^+^ CD45^−^ TER119^−^ hepatic progenitor cells. Left panels: dot plot of c-Kit and TER119/CD45. Right panels: dot plot of CD29 and CD49f. D: Absolute numbers of c-kit^−^ CD29^+^ CD49f^+^ CD45^−^ TER119^−^ hepatic progenitor cells per a liver of the control and Ezh2 KO at ED 13.5 (TAM; ED 8.5–10.5). Data are mean ± SD (n = 3). *P<0.05. E: Representing images of colonies derived from c-kit^−^ CD29^+^ CD49f^+^ CD45^−^ TER119^−^ cells at day 5 of culture obtained from the control and Ezh2 KO livers at ED 13.5 (TAM; ED 8.5–10.5) and their immunofluorescence labeling of Albumin (hepatocyte marker) and CK7 (cholangicoyte marker) positive cells. Scale bar = 200 µm. F: Number of hepatic colony-forming units in culture (H-CFU-C) derived colonies composed of over 90 cells at day 5 of culture. The c-kit^−^ CD29^+^ CD49f^+^ CD45^−^ TER119^−^ cells were freshly isolated from the control and Ezh2 KO fetal livers at ED 13.5 (TAM; ED 8.5–10.5) and underwent a clonal density colony formation assay. Data are mean ± SD (n = 3). *P<0.05. G: Average number of cells in each colony at day 5 of culture of c-kit^−^ CD29^+^ CD49f^+^ CD45^−^ TER119^−^ cells in the control and Ezh2 KO livers at ED 13.5 (TAM; ED 8.5–10.5). Data are mean ± SD (n = 3). *P<0.05. H: Number of bi-potent colonies in H-CFU-C derived colonies at day 5 of culture of c-kit^−^ CD29^+^ CD49f^+^ CD45^−^ TER119^−^ cells in the control and Ezh2 depleted. Data are mean ± SD (n = 3). *P<0.05. I: qRT-PCR analysis of expression levels of cyclin-dependent kinase inhibitor genes in the 5 day cultured c-kit^−^ CD29^+^ CD49f^+^ CD45^−^ TER119^−^ hepatic progenitor cells from the control and Ezh2 KO depletion livers at ED 13.5 (TAM; ED 8.5–10.5).

We hypothesized that the observed impairment of liver growth in size, weight, and number of cellular components upon Ezh2 SET domain depletion in the early embryonic livers might be caused by blockade of proliferation of hepatic progenitor cells. We first examined number of hepatic progenitor cells when Ezh2 SET domain was depleted at the early stage. An immunofluorescence analysis showed that frequency of AFP- and BrdU-positive proliferating cells were markedly decreased in the CreER(T2)^+/−^ Ezh2^F/F^ livers compared to control mice (Figures 4A and 4B). A flow-cytometric analysis showed that both proportional ratio and absolute number of c-kit^−^ CD49f^+/low^ CD29^+^ CD45^−^ TER119^−^ cells, that were identified as the hepatic progenitor population previously [3], was significantly reduced in CreER(T2)^+/−^ Ezh2^F/F^ livers compared to those in the control mice (Figure 4C and 4D). These results indicate that Ezh2 SET domain depletion inhibited the growth of hepatic progenitor cells *in vivo*. Next we sorted the c-kit^−^ CD49f^+/low^ CD29^+^ CD45^−^ TER119^−^ progenitor cells from CreER(T2)^+/−^ Ezh2^F/F^ and CreER(T2)^−/−^ Ezh2^F/F^ livers, and directly examined the effect of Ezh2 SET domain depletion on clonogenecity of the hepatic progenitor cells by *in vitro* clonal colony assay. After cultivating for 6 days, we could detect colonies containing both a hepatocyte marker, albumin- and a cholangiocyte marker, CK7-positive cells, indicating the bipotentiality of the sorted hepatic progenitor cells (Figure 4E). The size of colonies derived from CreER(T2)^+/−^ Ezh2^F/F^ progenitor cells was significantly smaller than that from the control cells (Figure 4E). A previously established quantitative assay for evaluation of clonogenic capacity of stem/progenitor cells, referred to as hepatic colony-forming units in culture (H-CFU-C) [3], demonstrated significant decrease of clonogenic potential of the Ezh2 SET domain deleted hepatic progenitor cells (Figure 4F). In addition average number of cells composing each colony was decreased by 60.0% in CreER(T2)^+/−^ Ezh2^F/F^ compared with the control (Figure 4G). Significant decrease in number of the bipotent colonies, which include both albumin and CK7 positive cells, was also observed (Figure 4H). These results indicate that Ezh2 SET domain depletion inhibits proliferation and clonogenicity of the hepatic progenitor cells, thereby resulting in impairment of differentiated liver cell generation. To gain insights into mechanisms underlying the inhibition of proliferation and clonogenicity of the hepatic progenitor cells, we carried out quantitative real-time PCR analysis on the cell cycle regulating genes. We confirmed that mRNA levels of the cyclin-dependent kinase inhibitor Cdkn1a, Cdkn2a, and Cdkn2b were significantly up-regulated upon Ezh2 SET domain depletion not only in the non-hematopoietic fetal liver cells (Figure 3A), but also in the c-kit^−^ CD49f^+/low^ CD29^+^ CD45^−^ TER119^−^ progenitor cell population (Figure 4I).

### Ezh2 SET domain depletion caused blockade of the cellular differentiation in embryonic livers

**Figure 5:**
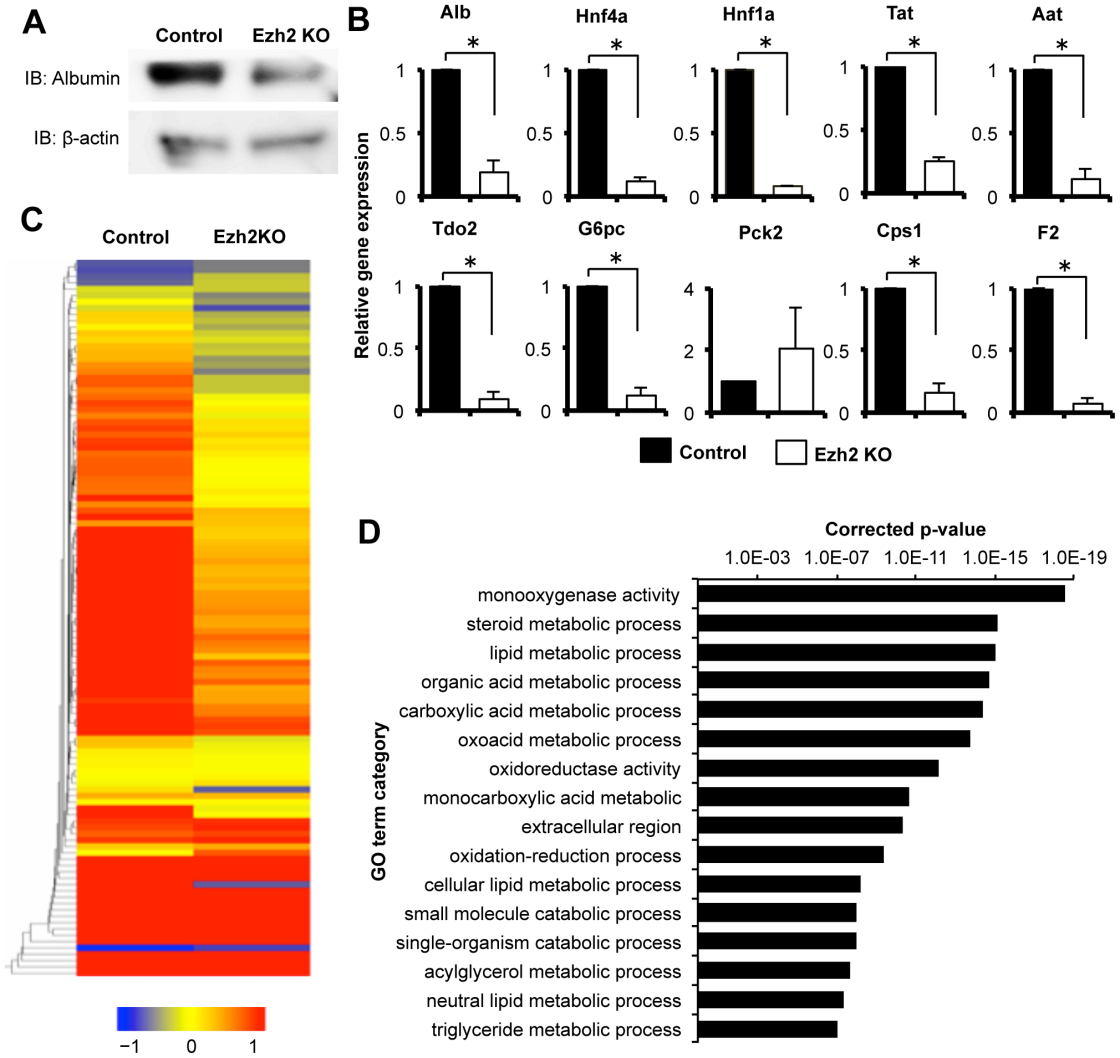
Ezh2 SET domain depletion caused blockade of the cellular differentiation in embryonic liver. A: Western blot analysis for Albumin (hepatocyte marker) in the CD45^−^ TER119^−^ non-hematopoietic liver cells from control (Rosa26::CreER(T2)^−/−^;Ezh2^F/F^ mouse) and Ezh2 KO (Ezh2 SET domain depleted mouse; Rosa26::CreER(T2)^+/−^;Ezh2^F/F^ mouse) at ED 18.5 after 3 days TAM injection (TAM; ED 10.5–12.5). B: Expression levels of hepatocytes related genes in the CD45^−^ TER119^−^ non-hematopoietic liver cells of the control and Ezh2 KO at indicated points were measured by qRT-PCR. Data are mean ± SD (n = 3). *P* values (asterisks) are from the Mann–Whitney *U*-test. *P<0.05. C: The heat map generated from microarray analysis for the liver development signature 83-gene expression in the CD45^−^ TER119^−^ non-hematopoietic liver cells of the control and Ezh2 KO. D: Significant enrichment of metabolism related liver functional GO terms for decreased genes upon Ezh2KO. Corrected *P* values of GO terms are shown.

We next examined the effects of the Ezh2 loss of function in the progression of cellular differentiation in the fetal livers. We first evaluated the liver at ED 13.5 when tamoxifen had been administered from ED 8.5. However, since the histological structure of the fetal liver tissue at ED 13.5 was still immature, the evaluation of differentiation based on histological analysis was difficult. For clear evaluation of the states of differentiation *in vivo*, we administered tamoxifen from ED 10.5, when the proliferating hepatic stem/progenitor cells still exist, and evaluated the liver structure at ED 18.5. Size of livers and composing parenchymal cell number were slightly decreased at ED 18.5 (data not shown). A western blot analysis demonstrated that expression of albumin was markedly decreased in CreER(T2)^+/−^ Ezh2^F/F^ liver cells compared to the control (Figure 5A). A qRT-PCR analysis showed that mRNA expression levels of hepatocyte specific genes: *Alb*, *Aat*, *Hnf4a*, *Hnf1a*, *Tat*, *Tdo2*, *G6pc*, *Cps1*, and *F2* were significantly decreased in the CreER(T2)^−/−^ Ezh2^F/F^ fetal liver cells compared to the control (Figure 5B), suggesting that Ezh2 SET domain depletion inhibited the differentiation to hepatocytes. Since hepatic progenitor cells differentiate into hepatocyte and cholangiocyte, we also examined the effect of Ezh2 SET domain depletion toward differentiation into cholangiocyte. HE staining showed that there was no significant difference in number of small ducts surrounding the large blood vessel, which is considered as bile duct or its precursor [21], but size of the ducts in CreER(T2)^+/−^ Ezh2^F/F^ liver was smaller than the control (Figures S2A and S2B). The mRNA expression levels of cholangiocyte marker genes: *Krt7*, *Krt19*, and *Hnf1b* in CreER(T2)^+/−^ Ezh2^F/F^ liver were less than that in CreER(T2)^−/−^ Ezh2^F/F^ fetal liver cells, although, we did not observe significance in the changes possibly due to rareness of the cholangiocyte population in frequency (Figure S2C). An immunofluorescence analysis confirmed that number of the CK7 positive cholangiocytes per a bile duct was decreased in CreER(T2)^+/−^ Ezh2^F/F^ liver (Figures S2D and S2E).

We next examined global gene expression profile for the liver parenchymal cells upon depletion of Ezh2 SET domain. We previously established a liver developmental gene signature that is composed of 83 genes, which are continuously up-regulated along with the progression of both human and mouse liver development, and are applicable for assessment of differentiation of liver-buds generated from iPS cells (Table S7) [20]. As expected, vast majority of the liver developmental gene signature was decreased by the depletion of the Ezh2 SET domain (Figure 5C). Moreover, the Gene Ontology (GO) Term Enrichment Analysis on the decreased genes by the Ezh2 loss of function identified a number of metabolic process related genes, such as steroid metabolic process, lipid metabolic process, and organic acid metabolic process as intensively enriched GO terms (Figure 5D and Table S8). These results demonstrate that progression of differentiation into functional hepatocytes is impaired by the Ezh2 SET domain depletion.

## Discussion

In this study, we show for the first time that Ezh2 function is essential for embryonic liver completion *in vivo*. The conditional depletion of SET domain of the *Ezh2* gene, which causes Ezh2 loss of function for catalytic induction of H3K27me3, results in significant reduction of liver in size. Our time-course expression analysis of the PRC1 and PRC2 component genes revealed that Ezh2 is expressed markedly higher in early stage, suggesting that Ezh2 would be a key PRC component that functions in the early stage. Concordantly, we observed more evident inhibitory effect of embryonic liver in particular when we induced Ezh2 loss of function from the early stage of ED 8.5. In the developing liver tissues, Ezh2 positive cells are densely enriched in the actively cycling immature hepatocytes, suggesting that Ezh2 would play a role in proliferating hepatic progenitor cells. Indeed, our clonal colony formation assay demonstrated that Ezh2 SET domain depletion abrogates clonogenicity of the directly isolated hepatic progenitor fraction from embryonic livers. These results also provide an explanation for the reduced number of the hepatic progenitor population and the total non-hematopoietic parenchymal cell population that consequently resulted in the reduced size of livers.

It was previously reported that cyclin-depedent kinase inhibitor 2A (Cdkn2a^ink4a/arf^) is a target gene of polycomb group proteins [22], [23]. For instance, Bmi1 controls proliferation of neural progenitor cells by repressing Cdkns including cdkn2a [24], [25]. We recently found that Ring1b, another component of PRC1, regulates the proliferation and self-renewal of hepatic progenitor cells through repressing both cdkn1a and cdkn2a [26]. Since it was also reported that Ezh2 regulates epidermal stem cells in skin by repressing Cdkn2a^ink4a/arf^ [27], Cdkn2a^ink4a/arf^ might be a potential target gene of Ezh2 for preventing from cell cycle arrest of the hepatic progenitor population. Indeed, our expression analysis identified multiple cell cycle regulating genes including Cdkn1a and Cdkn2a as significantly up-regulated genes upon Ezh2 SET domain depletion at the early liver development (Figure 3A). In addition, we confirmed accumulation of Ezh2 protein on the promoters of the Cdkn genes including Cdkn1a and 2a by a chromatin immunoprecipitation assay (Figure 3B). Further studies are needed for identification of target genes of Ezh2 that are responsible for the regulation of proliferation of the hepatic progenitor cells.

Among PcG proteins, an emerging body of evidence suggests that overexpression, amplification, and/or somatic mutation of Ezh2 are strongly involved in many types of cancer [28]. Recent studies showed that expression of Ezh2 is correlated with the worse outcome of patients with hepatocellular carcinoma (HCC), and regulates proliferation and tumorigenicity of tumor-spheres derived from HCC cell lines [29], [30]. Indeed, 3-Deazaneplanocin A (DZNep), which depletes PCR2 components and levels of H3K27me3 [30], inhibits proliferation of cancer stem cells of brain and breast cancer [31]–[33]. Our present data indicating an essential role of Ezh2 specifically for highly proliferating undifferentiated cells would imply that cancer cells might reacquire the similar properties of fetal progenitors. Further studies to clarify similarities and differences between hepatic progenitors and cancer cells may provide clues to develop better therapeutics against cancer.

We observed significant decrease of expression levels of hepatic differentiation markers together with numerous functional genes for liver upon Ezh2 loss of function, indicating that Ezh2 is required for normal liver maturation with accomplished cellular differentiation *in vivo*. A recent study reported that shRNA-mediated knockdown of Ezh2 in hepatic progenitor cells increased hepatic differentiation related genes *in vitro* [19]. This observation is contradictory to our present data that showed decreased differentiation in Ezh2 SET domain depleted liver. Although it is currently unknown what caused this discrepancy, one of possible explanations would be that Ezh2 might have caused temporal promotion of differentiation in hepatic progenitor cells so that led exhaustion of the progenitor pool required for establishment of the adequate cellular components of liver. Indeed, it was previously reported that exhaustion of stem and/or progenitor pools in hematopoietic and central nervous system caused incompletion of development [15], [16]. Alternatively, loss of function of Ezh2 that is independent of the SET domain might have mediated the enhancement of hepatic differentiation *in vitro*. In addition, it might reflect the technical difficulty in our assay to observe induction of differentiation in the freshly isolated hepatic progenitor cells from the fetal livers, of which proliferation is severely inhibited, while there would likely be more chances to see differentiation in the already established cell culture system.

For maintenance and identities of ES cells, it is shown that repression of development-regulating transcription factors by PRC2 is essential [34]. A repressive histone modification H3K27me3 accumulates at target loci together with activating histone modification of H3K4me3, forming so-called bivalent domains to keep the target genes poised [13]. Recently, it was reported that introduction of *Ezh2* is capable of enhancing efficiency to generate iPS cells and knockdown of Ezh2 attenuated the reprograming process, demonstrating direct role of Ezh2 for regulation of pluripotent stem cells [14]. It was also reported that Ezh1 functions as partially complement for Ezh2 to induce H3K27me3 in ES cells [12]. Given that Ezh2 loss of function solely caused severe blockade of liver growth and the very low expression level of Ezh1 in fetal liver, our findings suggest hepatic progenitor cells are dependent on Ezh2 for H3K27 tri-methylation activity. In line with this, similar Ezh2-dependency of fetal hematopoietic stem cells has been reported, when importance of Ezh1 was shown in adult hematopoietic stem cells [16], [18].

Recent advances made in the fields of stem cell biology and regenerative medicine highlight the potential application of tissue stem/progenitor cells for regenerative medicine. For patients with liver failure, intensified efforts have been made for establishment for complementation of liver function. We recently succeeded to generate functional liver buds having blood vessel networks and a three-dimensional tissue architecture from human iPSC-derived hepatic progenitor cells, which was facilitated by co-transplantation with mesenchymal stem cells and endothelial progenitor cells *in vivo* [20], [35]. In addition, generating abundant hepatocytes is urgently desired to survey adverse effects of newly developed drugs, as liver injury is a major reason that limits clinical usage of new drugs. For clinical application, however, such regenerative technologies are challenged by difficulty to obtain abundant hepatic progenitor cells. The present study underscores essential roles of Ezh2 for proliferation of hepatic progenitor cells and provides a basis for potential utilization of the Ezh2-mediated PRC2 machinery to efficiently enhance expansion of hepatic progenitor cells.

## Supporting Information

Figure S1 **Macroscopic images of livers of the control and Ezh2 KO at ED 13.5 (TAM; ED 8.5–10.5), in addition to** **Figure 2F** **.**
(TIF)

Figure S2 A: HE staining of liver tissues in the control and Ezh2 KO at ED 18.5 (TAM; ED 10.5–12.5). Arrows indicate bile ducts. Scale bar = 50 µm. B: Size of bile ducts (µm^2^) per sectioned area of liver at indicated point of the control and Ezh2 KO. Data are mean ± SD (n = 3). C: qRT-PCR analysis of expression levels of cholangiocyte related genes of the control and Ezh2 KO at indicated time points. Data are mean ± SD (n = 3). D: Immunofluorescence staining for CK7 and DAPI in liver tissues at an indicated point of the control and Ezh2 KO. Arrows indicate CK7 expressing cholangiocytes. PV: portal vein. Scale bar = 100 µm. E: Absolute number of CK7 positive cells per portal vein in sectioned area at indicated point. Data are mean ± SD (n = 3).
(TIF)

Table S1 **Genomic PCR primers used in this study.**
(DOCX)

Table S2 **Antibodies used in this study.**
(DOCX)

Table S3 **The quantitative reverse transcriptase PCR primers used in this study.**
(DOCX)

Table S4 **Chromatin Immunoprecipitation-PCR primers used in this study.**
(DOCX)

Table S5 **List of categories identified by the pathway analysis on significantly up-regulated genes by Ezh2 SET domain depletion (2-fold change).**
(DOCX)

Table S6 **List of categories identified by the pathway analysis on significantly up-regulated genes by Ezh2 SET domain depletion (8-fold change).**
(DOCX)

Table S7 **Liver developmental gene signatures was decreased by Ezh2 SET domain depletion.**
(DOCX)

Table S8 **Gene Ontology (GO) analyses of down-regulated genes by Ezh2 depletion.**
(DOCX)
