# Supplementary figures and images for: Polycomb Group Protein Ezh2 Regulates Hepatic Progenitor Cell Proliferation and Differentiation in Murine Embryonic Liver
Source: PLoS One. 2014 Aug 25;9(8):e104776. doi: 10.1371/journal.pone.0104776 (PMC4143191; doi:10.1371/journal.pone.0104776)

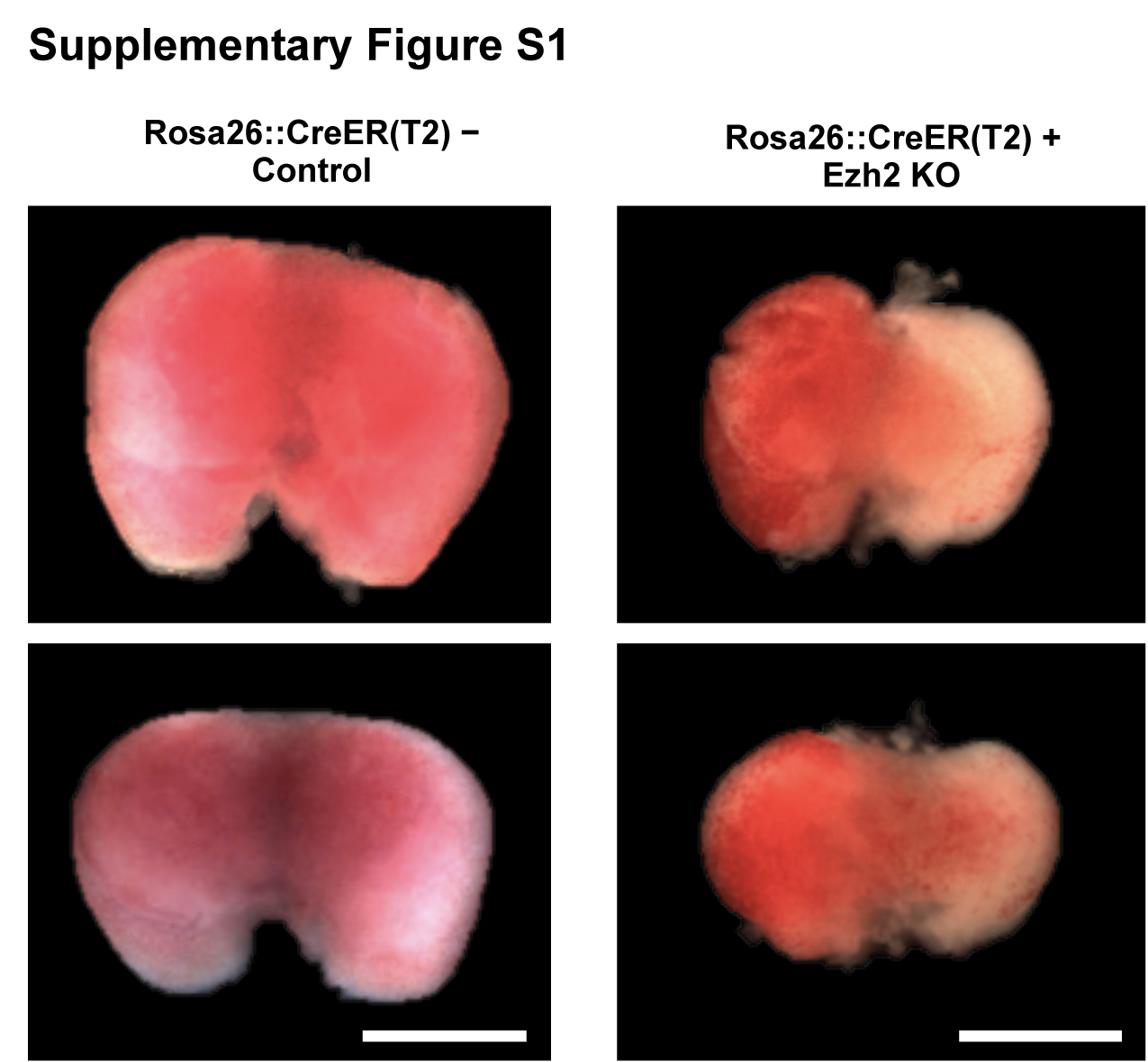

Supplement: Figure S1 — Macroscopic images of livers of the control and Ezh2 KO at ED 13.5 (TAM; ED 8.5–10.5), in addition to Figure 2F . (TIF) [file pone.0104776.s001.tif]

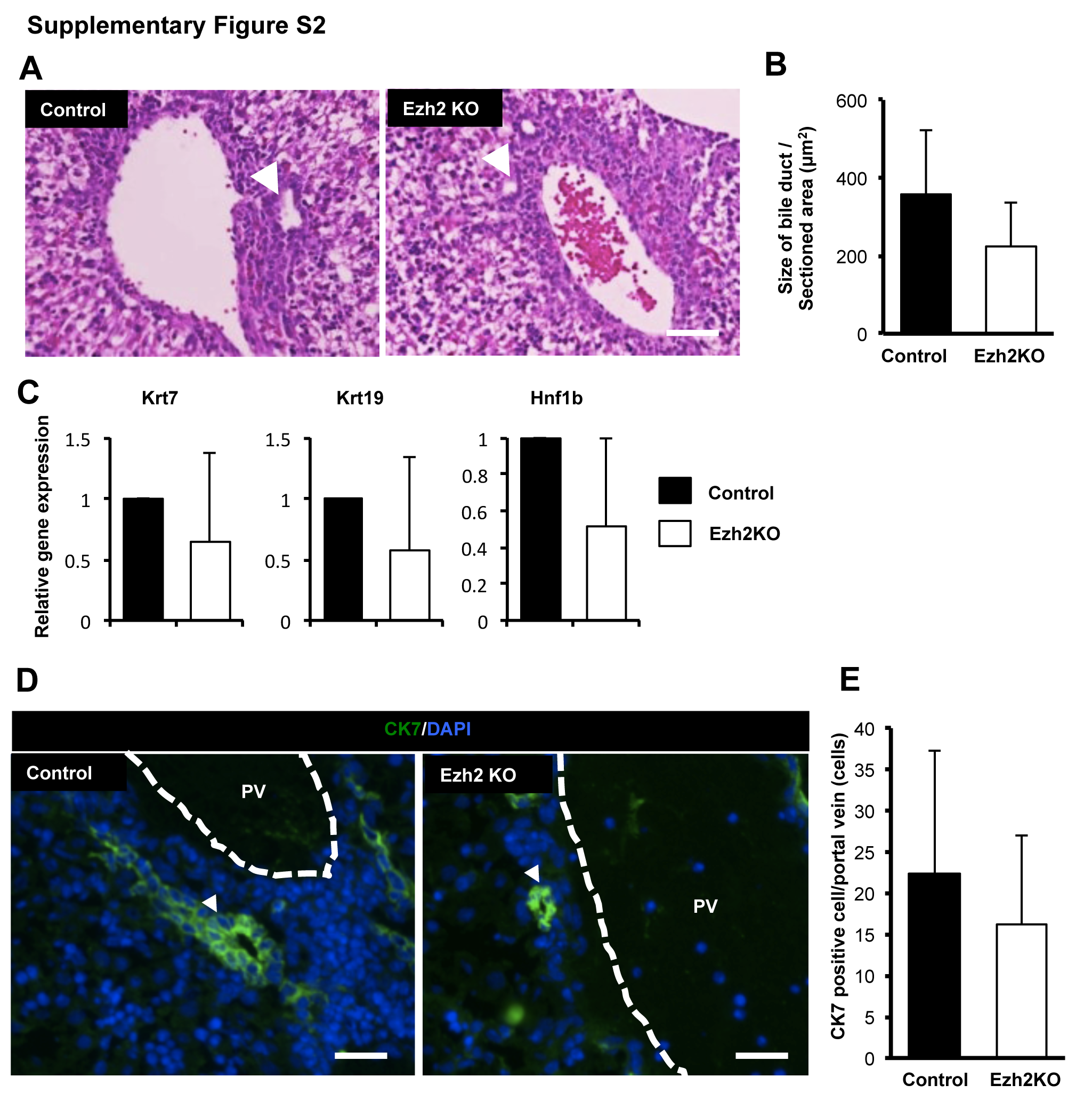

Supplement: Figure S2 — A: HE staining of liver tissues in the control and Ezh2 KO at ED 18.5 (TAM; ED 10.5–12.5). Arrows indicate bile ducts. Scale bar = 50 µm. B: Size of bile ducts (µm2) per sectioned area of liver at indicated point of the control and Ezh2 KO. Data are mean ± SD (n = 3). C: qRT-PCR analysis of expression levels of cholangiocyte related genes of the control and Ezh2 KO at indicated time points. Data are mean ± SD (n = 3). D: Immunofluorescence staining for CK7 and DAPI in liver tissues at an indicated point of the control and Ezh2 KO. Arrows indicate CK7 expressing cholangiocytes. PV: portal vein. Scale bar = 100 µm. E: Absolute number of CK7 positive cells per portal vein in sectioned area at indicated point. Data are mean ± SD (n = 3). (TIF) [file pone.0104776.s002.tif]
