# Supplementary material for: Polycomb Group Protein Ezh2 Regulates Hepatic Progenitor Cell Proliferation and Differentiation in Murine Embryonic Liver
Source: PLoS One. 2014 Aug 25;9(8):e104776. doi: 10.1371/journal.pone.0104776 (PMC4143191; doi:10.1371/journal.pone.0104776)
Supplement: Table S1 — Genomic PCR primers used in this study. (DOCX) [file pone.0104776.s003.docx]

**Supplementary Table S1. Genomic PCR primers used in this study.**

| Gene | | Forward | Reverse |
| --- | --- | --- | --- |
| Cre |  | CACCCTGTTACGTATAGCC | CTCTGACCAGAGTCATCCT |
| Ezh2 | F1  R1 | AAGGCTGTGTACAGGAAACAATC  ———————————————————————————————— | ————————————————————————————————  TCACCTTAATATGCGAAGTGGAC |
|  | R2 | ———————————————————————————————— | AGTACTCCAGAGGTACTGAAGTTTG |
