# Supplementary material for: Polycomb Group Protein Ezh2 Regulates Hepatic Progenitor Cell Proliferation and Differentiation in Murine Embryonic Liver
Source: PLoS One. 2014 Aug 25;9(8):e104776. doi: 10.1371/journal.pone.0104776 (PMC4143191; doi:10.1371/journal.pone.0104776)
Supplement: Table S2 — Antibodies used in this study. (DOCX) [file pone.0104776.s004.docx]

**Supplementary Table S2. Antibodies used in this study.**

| Target protein | Source |
| --- | --- |
| Cytokeratin 7 | Dako |
| Albumin | Biogenesis |
| α-Fetoprotein | MP bio |
| Cytokeratin 8/18 | Progen |
| BrdU | PharMingen |
| α1-antitrypsin | Santa Cruz |
| TER119 | PharMingen |
| CD45 | PharMingen |
| CD29 | PharMingen |
| CD49f | PharMingen |
| c-Kit | BioLegend |
| Ezh2 | Cell signaling |
| H3K27me3 | Millipore |
| Ring1B | MBL |
| Bmi1 | Millipore |
| Cdkn1a | DAKO |
| β-actin | SIGMA |
