# Supplementary material for: Polycomb Group Protein Ezh2 Regulates Hepatic Progenitor Cell Proliferation and Differentiation in Murine Embryonic Liver
Source: PLoS One. 2014 Aug 25;9(8):e104776. doi: 10.1371/journal.pone.0104776 (PMC4143191; doi:10.1371/journal.pone.0104776)
Supplement: Table S3 — The quantitative reverse transcriptase PCR primers used in this study. (DOCX) [file pone.0104776.s005.docx]

**Supplementary Table S3. The quantitative reverse transcriptase PCR primers used in this study.**

| Gene | Forward | Reverse |
| --- | --- | --- |
| *Albumin* | AGTGTTGTGCACACCTGAC | TTCTCCTTCACACCCATCAAGC |
| *Aat* | CATTGCTTAAATACAGACTAGGACAGG | AGATGGAGGGGAGTCATTTCAG |
| *Tat* | GGAGGAGGTCGCTTCCTATT | GCCACTCGTCAGAATGACATC |
| *Hnf4a*  *Hnf1a* | CCAAGAGGTCCATGGTGTTT  CGCCTCCACCCTGGTTAT | CCGAGGGACGATGTAGTCAT  ACTCCCCATGCTGTTGATG |
| *Tdo2* | TCCAGGGAGCACTGATGATA | CTGGAAAGGGACCTGGAATC |
| *G6pc* | TCTGTCCCGGATCTACCTTG | GAAAGTTTCAGCCACAGCAA |
| *Pck2* | CAGGGTCTTATCCGCAAACT | CACATCCTTGGGGTCTGTG |
| *Cps1* | CCAGTTTTGCAGTGGAATCA | GGTAGCCAATGGTGTCTGCT |
| *F2* | GCAGTGTCCCTGTCTGTGG | TTGTCCTTGGAACCTCCAGA |
| *Krt 19* | TGACCTGGAGATGCAGATTG | CCTCAGGGCAGTAATTTCCTC |
| *Krt 7* | GGAGATGGCCAACCACAG | GGCCTGGAGTGTCTCAAACTT |
| *Hnf1b* | ATGGCTCCCCTCACCATC | GGTTGTAGCGCACTCCTGA |
| *Gapdh* | AGCTTGTCATCAACGGGAAG | TTTGATGTTAGTGGGGTCTCG |
