# Supplementary material for: Polycomb Group Protein Ezh2 Regulates Hepatic Progenitor Cell Proliferation and Differentiation in Murine Embryonic Liver
Source: PLoS One. 2014 Aug 25;9(8):e104776. doi: 10.1371/journal.pone.0104776 (PMC4143191; doi:10.1371/journal.pone.0104776)
Supplement: Table S4 — Chromatin Immunoprecipitation-PCR primers used in this study. (DOCX) [file pone.0104776.s006.docx]

**Supplementary Table S4.** **Chromatin Immunoprecipitation-PCR primers used in this study.**

| Gene | Forward | Reverse |
| --- | --- | --- |
| *Cdkn2a* | TCGCACGATGTCTTGATGTC | TCAACTACGGTGCAGATTCG |
| *Cdkn1a* | AGCAAAGTGTGCCGTTGTC | TCAACTACGGTGCAGATTCG |
| *Cdkn2b* | TGCAGATACCTCGCAATGTC | TTACCAGACCTGTGCACGAC |
