# Supplementary material for: Polycomb Group Protein Ezh2 Regulates Hepatic Progenitor Cell Proliferation and Differentiation in Murine Embryonic Liver
Source: PLoS One. 2014 Aug 25;9(8):e104776. doi: 10.1371/journal.pone.0104776 (PMC4143191; doi:10.1371/journal.pone.0104776)
Supplement: Table S5 — List of categories identified by the pathway analysis on significantly up-regulated genes by Ezh2 SET domain depletion (2-fold change). (DOCX) [file pone.0104776.s007.docx]

| Pathway | p-value | Matched  Entities | Pathway Entities of  Experiment Type |
| --- | --- | --- | --- |
| Cell Receptor Signaling Pathway | 2.37E-04 | 35 | 133 |
| MAPK signaling pathway | 2.51E-04 | 40 | 159 |
| MicroRNAs in cardiomyocyte hypertrophy | 4.99E-04 | 24 | 104 |
| IL-5 Signaling Pathway | 5.19E-04 | 21 | 69 |
| G1 to S cell cycle control | 0.001870362 | 18 | 62 |
| IL-3 Signaling Pathway | 0.003645066 | 25 | 100 |
| Kit Receptor Signaling Pathway | 0.005683079 | 18 | 67 |
| Glycolysis and Gluconeogenesis | 0.006436885 | 14 | 48 |
| Senescence and Autophagy | 0.011019609 | 21 | 87 |
| Androgen Receptor Signaling Pathway | 0.014470516 | 25 | 112 |
| Toll Like Receptor signaling | 0.015099771 | 10 | 33 |
| Amino Acid metabolism | 0.01528723 | 22 | 95 |
| Insulin Signaling | 0.015800668 | 33 | 159 |
| IL-9 Signaling Pathway | 0.01595764 | 8 | 24 |
| EGFR1 Signaling Pathway | 0.016793154 | 36 | 176 |
| MAPK Cascade | 0.01767348 | 9 | 29 |
| Urea cycle and metabolism of amino groups | 0.017987618 | 7 | 20 |
| Proteasome Degradation | 0.018313045 | 15 | 59 |
| Fatty Acid Beta Oxidation | 0.018689584 | 10 | 34 |
| Cell cycle | 0.019208368 | 20 | 88 |
| miRNA regulation of DNA Damage Response | 0.020450683 | 16 | 91 |
| Wnt Signaling Pathway | 0.021247793 | 15 | 60 |
| Calcium Regulation in the Cardiac Cell | 0.023551418 | 31 | 150 |
| IL-6 signaling Pathway | 0.024076829 | 22 | 99 |
| Adipogenesis | 0.024159668 | 28 | 133 |
| estrogen signalling | 0.03264555 | 17 | 74 |
| metapathway biotransformation | 0.034836326 | 29 | 143 |
| Apoptosis Modulation by HSP70 | 0.035453074 | 6 | 18 |
| Iron Homeostasis | 0.040404063 | 5 | 14 |
| IL-2 Signaling Pathway | 0.041158695 | 17 | 76 |
| IL-4 signaling Pathway | 0.049752124 | 14 | 61 |

**Supplementary Table S5. List of categories identified by the pathway analysis on significantly up-regulated genes by Ezh2 SET domain depletion (2-fold change)**
