# Supplementary material for: Polycomb Group Protein Ezh2 Regulates Hepatic Progenitor Cell Proliferation and Differentiation in Murine Embryonic Liver
Source: PLoS One. 2014 Aug 25;9(8):e104776. doi: 10.1371/journal.pone.0104776 (PMC4143191; doi:10.1371/journal.pone.0104776)
Supplement: Table S6 — List of categories identified by the pathway analysis on significantly up-regulated genes by Ezh2 SET domain depletion (8-fold change). (DOCX) [file pone.0104776.s008.docx]

**Supplementary Table S6. List of categories identified by the pathway analysis on significantly up-regulated genes by Ezh2 SET domain depletion (8-fold change)**

| Pathway | p-value | Matched Entities | Pathway Entities of Experiment Type |
| --- | --- | --- | --- |
| Eicosanoid Synthesis | 1.46E-04 | 3 | 19 |
| IL-5 Signaling Pathway | 5.65E-04 | 4 | 69 |
| G1 to S cell cycle control | 0.004575472 | 3 | 62 |
| Inflammatory Response Pathway | 0.011676627 | 2 | 30 |
| TGF-beta Receptor Signaling Pathway | 0.049388513 | 3 | 150 |
