# Supplementary material for: Polycomb Group Protein Ezh2 Regulates Hepatic Progenitor Cell Proliferation and Differentiation in Murine Embryonic Liver
Source: PLoS One. 2014 Aug 25;9(8):e104776. doi: 10.1371/journal.pone.0104776 (PMC4143191; doi:10.1371/journal.pone.0104776)
Supplement: Table S7 — Liver developmental gene signatures was decreased by Ezh2 SET domain depletion. (DOCX) [file pone.0104776.s009.docx]

**Supplementary Table S7. Liver developmental gene signatures was decreased by Ezh2 SET domain depletion**

| Gene Name | Gene  Symbol | Regulation (Ezh2KO vs WT) | Fold Change (Ezh2KO vs WT) |
| --- | --- | --- | --- |
| claudin 2 | Cldn2 | down | 4.3474736 |
| histidine-rich glycoprotein | Hrg | down | 2.8470242 |
| dihydropyrimidinase | Dpys | down | 2.558137 |
| flavin containing monooxygenase 5 | Fmo5 | down | 2.2173245 |
| 3-hydroxy-3-methylglutaryl-Coenzyme A synthase 2 | Hmgcs2 | down | 2.1439939 |
| coagulation factor XII (Hageman factor) | F12 | down | 2.0535884 |
| leucine-rich alpha-2-glycoprotein 1 | Lrg1 | down | 2.0529435 |
| apolipoprotein C-III | Apoc3 | down | 2.0363436 |
| methionine adenosyltransferase I, alpha | Mat1a | down | 1.9691765 |
| complement component factor h | Cfh | down | 1.968252 |
| 3-hydroxy-3-methylglutaryl-Coenzyme A synthase 2 | Hmgcs2 | down | 1.9514315 |
| complement component factor h | Cfh | down | 1.8940638 |
| ATP-binding cassette, sub-family A (ABC1), member 6 | Abca6 | down | 1.8860714 |
| haptoglobin | Hp | down | 1.8837514 |
| cytochrome P450, family 27, subfamily a, polypeptide 1 | Cyp27a1 | down | 1.858502 |
| fructose bisphosphatase 1 | Fbp1 | down | 1.8550628 |
| epoxide hydrolase 2, cytoplasmic | Ephx2 | down | 1.853299 |
| serine (or cysteine) peptidase inhibitor, clade G, member 1 | Serping1 | down | 1.8467978 |
| flavin containing monooxygenase 5 | Fmo5 | down | 1.8461888 |
| homogentisate 1, 2-dioxygenase | Hgd | down | 1.8450395 |
| serine (or cysteine) peptidase inhibitor, clade G, member 1 | Serping1 | down | 1.821626 |
| inter alpha-trypsin inhibitor, heavy chain 4 | Itih4 | down | 1.810592 |
| formiminotransferase cyclodeaminase | Ftcd | down | 1.8007495 |
| kininogen 1 | Kng1 | down | 1.7997416 |
| ectonucleoside triphosphate diphosphohydrolase 8 | Entpd8 | down | 1.7995833 |
| defensin beta 1 | Defb1 | down | 1.7995375 |
| complement component 3 | C3 | down | 1.7758225 |
| inter alpha-trypsin inhibitor, heavy chain 4 | Itih4 | down | 1.7635138 |
| kininogen 1 | Kng1 | down | 1.7553248 |
| enoyl Coenzyme A hydratase domain containing 2 | Echdc2 | down | 1.7380104 |
| angiogenin, ribonuclease, RNase A family, 5 | Ang | down | 1.7319674 |
| acyl-Coenzyme A oxidase 1, palmitoyl | Acox1 | down | 1.7276417 |
| plasminogen | Plg | down | 1.7250068 |
| dihydropyrimidinase | Dpys | down | 1.7079936 |
| complement component 3 | C3 | down | 1.6903415 |
| flavin containing monooxygenase 5 | Fmo5 | down | 1.6878326 |
| ethanolamine kinase 2 | Etnk2 | down | 1.6871008 |
| aldolase B, fructose-bisphosphate | Aldob | down | 1.6857017 |
| sarcosine dehydrogenase | Sardh | down | 1.6729623 |
| 3-hydroxyanthranilate 3,4-dioxygenase | Haao | down | 1.6714944 |
| orosomucoid 1 | Orm1 | down | 1.6604019 |
| complement factor B | Cfb | down | 1.6429565 |
| enoyl Coenzyme A hydratase domain containing 2 | Echdc2 | down | 1.6300982 |
| aldehyde dehydrogenase family 3, subfamily A2 | Aldh3a2 | down | 1.6260678 |
| dihydroxyacetone kinase 2 homolog (yeast) | Dak | down | 1.6234043 |
| acetyl-Coenzyme A acetyltransferase 1 | Acat1 | down | 1.581142 |
| dicarbonyl L-xylulose reductase | Dcxr | down | 1.5775046 |
| complement component factor i | Cfi | down | 1.5746021 |
| sterol carrier protein 2, liver | Scp2 | down | 1.5697467 |
| glyoxylate reductase/hydroxypyruvate reductase | Grhpr | down | 1.5678986 |
| glyoxylate reductase/hydroxypyruvate reductase | Grhpr | down | 1.56591 |
| G0/G1 switch gene 2 | G0s2 | down | 1.5392053 |
| hydroxy-delta-5-steroid dehydrogenase, 3 beta- and steroid delta-isomerase 7 | Hsd3b7 | down | 1.537253 |
| fibronectin 1 | Fn1 | down | 1.5285943 |
| FGGY carbohydrate kinase domain containing | Fggy | down | 1.5122342 |
| ubiquitin D | Ubd | down | 1.5089494 |
| FGGY carbohydrate kinase domain containing | Fggy | down | 1.505632 |
| dicarbonyl L-xylulose reductase | Dcxr | down | 1.5053469 |
| retinoic acid receptor responder (tazarotene induced) 2 | Rarres2 | down | 1.5052927 |
| coiled-coil-helix-coiled-coil-helix domain containing 10 | Chchd10 | down | 1.4971424 |
| claudin 2 | Cldn2 | down | 1.4833496 |
| FGGY carbohydrate kinase domain containing | Fggy | down | 1.4604089 |
| patatin-like phospholipase domain containing 7 | Pnpla7 | down | 1.4545752 |
| orosomucoid 2 | Orm2 | down | 1.4532672 |
| START domain containing 10 | Stard10 | down | 1.4413626 |
| ethanolamine kinase 2 | Etnk2 | down | 1.4410897 |
| vitamin K epoxide reductase complex, subunit 1 | Vkorc1 | down | 1.4290153 |
| receptor accessory protein 6 | Reep6 | down | 1.4241191 |
| FGGY carbohydrate kinase domain containing | Fggy | down | 1.4200891 |
| regucalcin | Rgn | down | 1.419876 |
| ethanolamine kinase 2 | Etnk2 | down | 1.4181876 |
| MAGI family member, X-linked | Magix | down | 1.4074423 |
| receptor accessory protein 6 | Reep6 | down | 1.3950207 |
| sterol carrier protein 2, liver | Scp2 | down | 1.3898368 |
| signal transducing adaptor family member 2 | Stap2 | down | 1.360648 |
| vitamin K epoxide reductase complex, subunit 1 | Vkorc1 | down | 1.3536693 |
| MAGI family member, X-linked | Magix | down | 1.3445954 |
| peroxisomal trans-2-enoyl-CoA reductase | Pecr | down | 1.3411685 |
| solute carrier family 16 (monocarboxylic acid transporters), member 2 | Slc16a2 | down | 1.341087 |
| insulin-like growth factor binding protein 4 | Igfbp4 | down | 1.3340083 |
| aldehyde dehydrogenase 2, mitochondrial | Aldh2 | down | 1.322334 |
| apolipoprotein C-III | Apoc3 | down | 1.3108667 |
| sorbitol dehydrogenase | Sord | down | 1.3105565 |
| electron transferring flavoprotein, dehydrogenase | Etfdh | down | 1.307222 |
| dehydrogenase E1 and transketolase domain containing 1 | Dhtkd1 | down | 1.2681583 |
| interleukin 13 receptor, alpha 1 | Il13ra1 | down | 1.2601936 |
| cathepsin O | Ctso | down | 1.2307243 |
| Kruppel-like factor 9 | Klf9 | down | 1.2185478 |
| peroxisomal membrane protein 2 | Pxmp2 | down | 1.2136041 |
| monoglyceride lipase | Mgll | down | 1.1201682 |
| aminolevulinic acid synthase 1 | Alas1 | down | 1.1071067 |
| nuclear factor I/C | Nfic | down | 1.0655572 |
| dehydrogenase/reductase (SDR family) member 1 | Dhrs1 | down | 1.0574317 |
| tumor necrosis factor (ligand) superfamily, member 10 | Tnfsf10 | down | 1.0522603 |
| monoglyceride lipase | Mgll | down | 1.0459436 |
| monoglyceride lipase | Mgll | down | 1.0291045 |
| nuclear factor I/C | Nfic | down | 1.0197533 |
| myxovirus (influenza virus) resistance 2 | Mx2 | down | 1.0115538 |
| hepcidin antimicrobial peptide | Hamp | up | 3.180861 |
| ATP-binding cassette, sub-family C (CFTR/MRP), member 3 | Abcc3 | up | 1.5805395 |
| interferon-induced protein with tetratricopeptide repeats 1 | Ifit1 | up | 1.3304874 |
| carboxylesterase 2H | Ces2h | up | 1.2790452 |
| proteasome (prosome, macropain) subunit, beta type 9 (large multifunctional peptidase 2) | Psmb9 | up | 1.2718661 |
| proteasome (prosome, macropain) subunit, beta type 9 (large multifunctional peptidase 2) | Psmb9 | up | 1.134041 |
| sphingomyelin phosphodiesterase, acid-like 3A | Smpdl3a | up | 1.1327007 |
| tryptophan 2,3-dioxygenase | Tdo2 | up | 1.1214781 |
| aminolevulinic acid synthase 1 | Alas1 | up | 1.093875 |
| peroxisomal trans-2-enoyl-CoA reductase | Pecr | up | 1.0812688 |
| interferon regulatory factor 7 | Irf7 | up | 1.0656452 |
| proteasome (prosome, macropain) subunit, beta type 8 (large multifunctional peptidase 7) | Psmb8 | up | 1.0652438 |
| nuclear factor I/C | Nfic | up | 1.0515673 |
| transporter 1, ATP-binding cassette, sub-family B (MDR/TAP) | Tap1 | up | 1.0194938 |
| monoglyceride lipase | Mgll | up | 1.0028087 |
