# Supplementary material for: Polycomb Group Protein Ezh2 Regulates Hepatic Progenitor Cell Proliferation and Differentiation in Murine Embryonic Liver
Source: PLoS One. 2014 Aug 25;9(8):e104776. doi: 10.1371/journal.pone.0104776 (PMC4143191; doi:10.1371/journal.pone.0104776)
Supplement: Table S8 — Gene Ontology (GO) analyses of down-regulated genes by Ezh2 depletion. (DOCX) [file pone.0104776.s010.docx]

**Supplementary Table S8. Gene Ontology (GO) analyses of down-regulated genes by Ezh2 depletion**

| ID | Term | # Genes in  Reference | # Genes in  Input | Corrected  P-value |
| --- | --- | --- | --- | --- |
| #GO:0004497  #GO:0008202  #GO:0006629  #GO:0006082  #GO:0019752  #GO:0043436  #GO:0016491  #GO:0032787  #GO:0005576  #GO:0055114  #GO:0044255  #GO:0044282  #GO:0044712  #GO:0006639  #GO:0006638  #GO:0006641 | monooxygenase activity  steroid metabolic process  lipid metabolic process  organic acid metabolic process  carboxylic acid metabolic process  oxoacid metabolic process  oxidoreductase activity  monocarboxylic acid metabolic  extracellular region  oxidation-reduction process  cellular lipid metabolic process  small molecule catabolic process  single-organism catabolic process  acylglycerol metabolic process  neutral lipid metabolic process  triglyceride metabolic process | \| 123 \| \| --- \| \| 190 \| \| 796 \| \| 646 \| \| 596 \| \| 632 \| \| 703 \| \| 324 \| \| 1655 \| \| 832 \| \| 596 \| \| 154 \| \| 154 \| \| 77 \| \| 79 \| \| 68 \| | \| 55 \| \| --- \| \| 59 \| \| 125 \| \| 45 \| \| 45 \| \| 45 \| \| 142 \| \| 32 \| \| 278 \| \| 150 \| \| 56 \| \| 1 \| \| 1 \| \| 27 \| \| 27 \| \| 27 \| | \| 3.09E-19 \| \| --- \| \| 7.15E-16 \| \| 1.05E-15 \| \| 2.12E-15 \| \| 3.79E-15 \| \| 1.83E-14 \| \| 6.84E-13 \| \| 2.28E-11 \| \| 4.39E-11 \| \| 3.90E-10 \| \| 5.84E-09 \| \| 1.10E-08 \| \| 1.10E-08 \| \| 2.26E-08 \| \| 4.83E-08 \| \| 9.12E-08 \| |
